# Biological conversion of aromatic monolignol compounds by a *Pseudomonas* isolate from sediments of the Baltic Sea

**DOI:** 10.1186/s13568-018-0563-x

**Published:** 2018-03-02

**Authors:** Krithika Ravi, Javier García-Hidalgo, Matthias Nöbel, Marie F. Gorwa-Grauslund, Gunnar Lidén

**Affiliations:** 1 Department of Chemical Engineering, Lund University, P.O. Box 124, 221 00 Lund, Sweden; 2 Department of Chemistry, Applied Microbiology, Lund University, P.O. Box 124, 221 00 Lund, Sweden

**Keywords:** Sediment isolates, *Pseudomonas*, Bacterial aromatic catabolism, Monolignols, Vanillyl alcohol

## Abstract

Bacterial strains were isolated from the sediments of the Baltic Sea using ferulic acid, guaiacol or a lignin-rich softwood waste stream as substrate. In total nine isolates were obtained, five on ferulic acid, two on guaiacol and two on a lignin-rich softwood stream as a carbon source. Three of the isolates were found to be *Pseudomonas* sp. based on 16S rRNA sequencing. Among them, isolate 9.1, which showed the fastest growth in defined M9 medium, was tentatively identified as a *Pseudomonas deceptionensis* strain based on the *gyrB* sequencing. The growth of isolate 9.1 was further examined on six selected lignin model compounds (ferulate, *p*-coumarate, benzoate, syringate, vanillin and guaiacol) from different upper funneling aromatic pathways and was found able to grow on four out of these six compounds. No growth was detected on syringate and guaiacol. The highest specific growth and uptake rates were observed for benzoate (0.3 h^−1^ and 4.2 mmol g_CDW_^−1^ h^−1^) whereas the lowest were for the compounds from the coniferyl branch. Interestingly, several pathway intermediates were excreted during batch growth. Vanillyl alcohol was found to be excreted during growth on vanillin. Several other intermediates like *cis,cis*-muconate, catechol, vanillate and 4-hydroxybenzoate from the known bacterial catabolic pathways were excreted during growth on the model compounds.

**Electronic supplementary material:**

The online version of this article (10.1186/s13568-018-0563-x) contains supplementary material, which is available to authorized users.

## Introduction

Lignin is one of the most plentiful biopolymers on Earth. It is a complex alkyl–aromatic heteropolymer found in the plant cell wall, which provides strength and protection to terrestrial plants. Currently, technical lignin is produced from either the pulp/paper industries and/or cellulosic ethanol biorefineries, where it is mainly used as an energy source to produce steam and electricity. Lignin is underexploited as a chemical feedstock because of its heterogeneity and intractable structure (Ayyachamy et al. 2013). The valorization of lignin to produce renewable fuels and chemicals is important to further develop the biorefineries (Beckham et al. 2016; Camarero et al. 2014; Rodriguez et al. 2017). To allow lignin to be used as a substrate for bioconversion, a depolymerization step is essential to generate a combination of monomeric, dimeric and oligomeric lignin-based compounds (Rinaldi et al. 2016; Xu et al. 2014). These can be further catabolized into various bio-based compounds of economic value for lignocellulosic biorefineries (Abdelaziz et al. 2016; Salvachúa et al. 2015).

Lignin in nature is degraded mainly by extracellular peroxidases and laccases, secreted by white-rot and brown-rot fungi. The action of these enzymes on lignin results in a diverse range of low molecular weight aromatic fragments (Martínez et al. 2005). Due to the widespread availability of these aromatic molecules in the environment, several microbes have developed catabolic pathways for these (Makela et al. 2015). There have been many studies on a variety of fungal species, which can utilize lignin-related compounds with the help of redox mediators (Martínez et al. 2005). However, their commercial application is limited by slow growth rates and difficulties related to fungal genetic manipulation (Bugg et al. 2011a).

In addition to fungi, there are several bacterial species capable of catabolizing lignin or lignin-related aromatic compounds (Brown and Chang 2014; Bugg et al. 2011b). Bacteria have certain benefits over fungi, e.g. easy genetic engineering, fast growth and ease of cultivation. Reported bacteria with ability to convert aromatics include *Pseudomonas putida* KT2440 (Jiménez et al. 2002; Ravi et al. 2017), *Cupriavidus necator* JMP134 (Pérez-Pantoja et al. 2008), *Rhodococcus opacus* (Zhao et al. 2016), *Rhodococcus jostii* RHA1 (Ahmad et al. 2011), *Acinetobacter baylyi* ADP1 (Barbe et al. 2004), *Amycolatopsis* sp. 75iv2 (Brown et al. 2011), *Sphingomonas* sp. strain SYK-6 (Masai et al. 2007) and *Streptomyces viridosporus* T7A (Ramachandra et al. 1988). Most likely, there are other bacteria with aromatic metabolizing capacities, which are yet to be identified. Recently, there has been an extensive search for more bacterial species from several natural or man-made environments, which exhibit certain lignin-degrading abilities thanks to the secretion of oxidoreductases, etherases and other enzymes (Picart et al. 2016; Taylor et al. 2012). Some of the reported organisms are, e.g. *Klebsiella* and *Pseudomonas* spp. from compost samples (Ravi et al. 2017), *Cupriavidus basilensis* B-8 (Shi et al. 2013) and *Comamonas* sp. B-9 from eroded bamboo slips (Chen et al. 2012), *Bacillus pumilus* and *Bacillus atrophaeus* from biodiversity-rich rainforest soil (Huang et al. 2013) and *Trabulsiella* sp. isolated from termite gut (Suman et al. 2016). It is essential not only to isolate these organisms, but also to characterize their inherent aromatic metabolism in order to assess their potential—as hosts or donor of pathways—for lignin valorization.

**Fig. 1:**
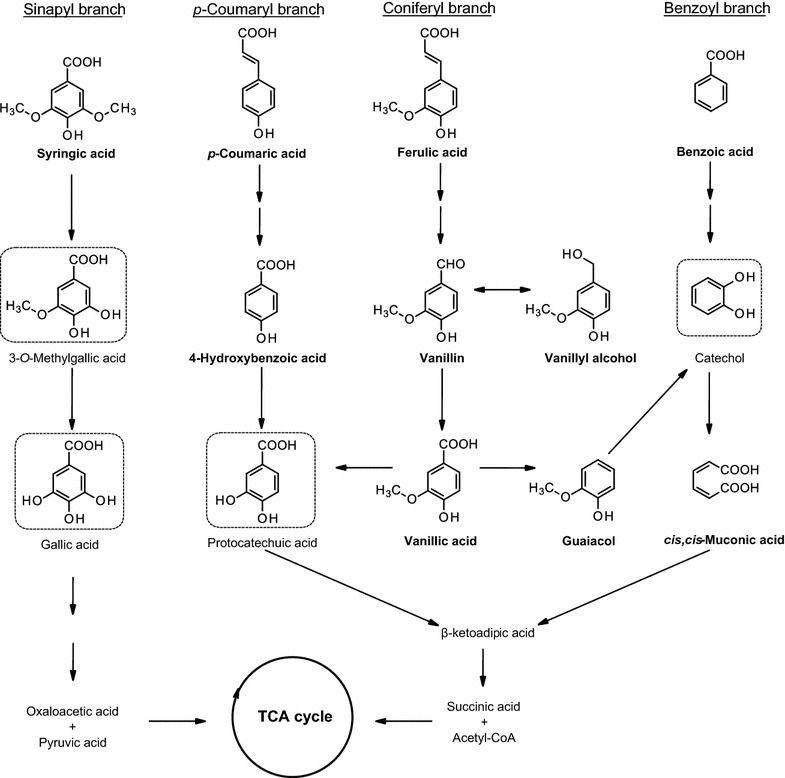
Major upper funneling pathways for the bacterial metabolism of lignin model compounds. The model compounds used in this study are indicated in bold. Metabolic intermediates that can be subjected to aromatic ring cleavage are indicated in dashed boxes

In the present study, our initial aim was to isolate and identify easily culturable bacterial species from sediments in the Baltic Sea, close to the wastewater stream of a sulfite pulp production plant in northern Sweden (Kramfors). In this environment, lignin-rich residuals were deposited and accumulated between 1907 and 1977 (Apler et al. 2014). Lignin-enriched wastewater effluents have a damaging effect to the aquatic ecosystems because of their toxic chemical compounds and also due to the recalcitrance of this polymer in natural environments (Berryman et al. 2004). Therefore bacterial species present in such polluted sediments are likely to possess aromatic metabolic capacities, and hence are interesting to investigate (Priyadarshinee et al. 2016). The second objective was to further examine the metabolism of aromatic compounds by the most interesting isolates. In particular, one of the isolates, identified as *Pseudomonas* sp. 9.1 was grown on six lignin model compounds (ferulate, *p*-coumarate, benzoate, syringate, vanillin and guaiacol) representing the main branches of the upper funneling catabolic pathways (Fig. 1) and specific growth rates, specific uptake rates and by-product formation from the aromatic compounds were quantified. The selected isolate, under the same initial aromatic compounds concentration, was found to behave quite differently from the previously studied *P. putida* KT2440 (Ravi et al. 2017) since it excreted several pathway intermediates. Further experiments were conducted with the excreted intermediates and the conversion rates of various upper funneling branches leading to β-ketoadipate pathway were assessed.

## Materials and methods

### Cultivation media

M9 mineral medium was used for all the experiments (Sambrook and Russell 2001). The composition is as follows: 6 g L^−1^ disodium phosphate, 3 g L^−1^ monopotassium phosphate, 0.5 g L^−1^ sodium chloride, 1 g L^−1^ ammonium chloride, 2 mM magnesium sulfate, 100 µM calcium chloride with 10 mL L^−1^ trace elements solution (Pfennig and Lippert 1966). The pH of the final medium was adjusted to 7. All the chemicals and materials used were purchased from Sigma-Aldrich (St. Louis, USA) or VWR (West Chester, PA, USA). Sterile conditions were maintained by either autoclavation or sterile filtration.

### Strains

In this study, nine bacterial strains were isolated from sediments of the Baltic Sea. The isolate used in the detailed characterization experiments, namely *Pseudomonas* sp. 9.1 was deposited in the DSMZ (German Collection of Microorganisms and Cell Cultures), with accession code DSM 105530. All the strains isolated in this work (see Table 1) are available for research purposes upon request.

**Table 1:** Identification of the bacterial isolates by 16S rRNA sequencing

Isolate	BLAST			EzBioCloud			Taxonomic group	Gram	Isolated from
	Species	Query cover (%)	Identity (%)	Species	Similarity (%)	Completeness (%)			
8.1	*Pseudomonas donghuensis* HYS (T)	100	98	*Pseudomonas alcaligenes* TPID2/JYOC 5	98.86	100	*γ*-*proteobacteria*, Pseudomonads	–	Ferulic acid
	*Pseudomonas alkylphenolia* KL28 (T)	100	98	*Pseudomonas alkylphenolia* KL28 (T)	98.37	100			
	*Pseudomonas lutea* OK2 (T)	100	98	*Pseudomonas donghuensis* HYS (T)	98.37	100			
8.2	*Lelliottia amnigena* JCM1237 (T)	99	99	*Lelliottia amnigena* NBRC 105700 (T)	99.72	100	*γ*-*proteobacteria, Enterobacteriaceae*	–	
	*Kluyvera intermedia* 256	99	99	*Kluyvera intermedia* ATCC 33110 (T)	98.86	100			
	*Kluyvera intermedia* NBRC 102594 (T)	99	99	*Raoultella terrigena* ATCC 33257 (T)	98.86	99.3			
9.1	*Pseudomonas deceptionensis* M1 (T)	99	99	*Pseudomonas weihenstephanensis* DSM29166 (T)	99.93	100	*γ*-*proteobacteria*, Pseudomonads	–	
	*Pseudomonas fragi* ATCC 4973	99	99	*Pseudomonas deceptionensis* M1 (T)	99.79	100			
	*Pseudomonas psychrophila* E-3 (T)	99	99	*Pseudomonas psychrophila* E-3 (T)	99.64	100			
9.2	*Pseudomonas umsongensis* Ps 3-10 (T)	99	99	*Pseudomonas moorei* RW10 (T)	99.78	100		–	
	*Pseudomonas baetica* a390 (T)	99	99	*Pseudomonas mohni*i Ipa-2 (T)	99.64	100			
	*Pseudomonas vancouverensis* DhA-51 (T)	99	99	*Pseudomonas umsongensis* Ps 3-10 (T)	99.58	99.7			
19	*Nocardia coeliaca* DSM 44595 (T)	99	99	*Rhodococcus erythropolis* NBRC 15567 (T)	99.71	100	*Actinobacteria, Nocardiaceae*	+	
	*Rhodococcus erythropolis* N11	99	99	*Rhodococcus qingshengii* JCM 15477 (T)	99.07	100			
	*Rhodococcus jialingiae* djl-6-2 (T)	99	99	*Rhodococcus degradans* CCM 4446 (T)	99.07	100			
47.1	*Acinetobacter lwoffii* DSM 2403 (T)	99	99	*Acinetobacter lwoffii* NIPH 715	98.87	100	*γ*-*proteobacteria*, Pseudomonads, *Moraxellaceae*	–	Guaiacol
	*Acinetobacter albensis* ANC 4874 (T)	99	99	*Acinetobacter lwoffii* NCTC 5866 (T)	98.58	100			
	*Prolinoborus fasciculus* CIP 103579 (T)	98	99	*Acinetobacter albensis* ANC 4874	98.44	100			
47.2	*Lysinibacillus macroides* LMG 18474 (T)	100	99	*Lysinibacillus macroides* DSM 54 (T)	99.58	100	*Firmicutes, Bacillaceae*	+	
	*Lysinibacillus boronitolerans* NBRC 103108 (T)	100	99	*Lysinibacillus xylanilyticus* DSM 23493 (T)	99.37	100			
	*Lysinibacillus pakistanensis* NCCP-54 (T)	99	99	*Lysinibacillus pakistanensis* JCM 18776 (T)	99.30	100			
49	*Bacillus licheniformis* DSM 13 (T)	100	99	*Bacillus licheniformis* ATCC 14580 (T)	99.76	100		+	Softwood stream
	*Bacillus sonorensis* NBRC 101234 (T)	100	99	*Bacillus paralicheniformis* KJ-16 (T)	99.76	86.4			
	*Bacillus aerius* 24 K (T)	100	99	*Bacillus sonorensis* NBRC 101234 (T)	99.61	100			
3B	*Bacillus safensis* NBRC 100820 (T)	100	99	*Bacillus safensis* FO-36b (T)	100	100		+	
	*Bacillus pumilus* NBRC 12092 (T)	100	99	*Bacillus australimaris* NH7I_1 (T)	99.86	100			
	*Bacillus aerius* 24 K (T)	100	99	*Bacillus* zhangzhouensis DW5-4 (T)	99.79	100			

Type strains are indicated with (T)

### Isolation and identification of bacterial strains from the Baltic Sea sediments

An enrichment culture protocol was developed with the purpose of isolating mesophilic culturable bacteria from Baltic Sea sediments. These fiber-rich sediments were obtained in the vicinity of a pulp and paper mill located in the northern Baltic coast of Sweden. The sludgy sediment sample (approximately 1 g) was initially washed with 4 mL of 0.8% sterile NaCl solution by vigorous vortexing, and after 5 min of settling the supernatant was used to inoculate culture plates. As a first stage prior to the screening with model compounds, a revitalization step in nutrient agar plates was included to allow dormant bacteria to revive after a freezing period. In order to enrich exclusively the bacteria present in this environment, cycloheximide was added to the screening cultures to inhibit the growth of fungi or other eukaryotes present in the sample. A number of different bacterial species were isolated by successive streaking on mineral M9 medium plates supplemented with different lignin-related model compounds, namely ferulic acid and guaiacol or lignin-rich softwood pulping streams. Isolates were grown in liquid LB medium or nutrient broth overnight prior to freezing and storage at − 80 °C.

Identification of the bacterial isolates was carried out by PCR amplification and sequencing of the 16S rRNA using genomic DNA of the isolates as template, exactly as described in Ravi et al. 2017. Further taxonomic study of the isolates belonging to the genus *Pseudomonas* was done by partial *gyrB* gene PCR amplification, using Phusion Hot Start II High-Fidelity DNA Polymerase (Thermo Scientific), the degenerate primers gyrB Fw (5′ GGGAACAGACBTACGTBCACGGYGTT 3′) and gyrB Rv (5′ GCTTTACGBGCSGCYTCACG 3′) and following this PCR program: Initial denaturation at 98 °C for 7 min, 30 cycles of: (denaturation at 98 °C for 10 s, annealing at 67 °C for 20 s, extension at 72 °C for 16 s) and final extension at 72 °C for 5 min. Amplified DNA fragments of around 700 bp were purified with GeneJET PCR Purification Kit (Thermo Scientific) and sent for sequencing with the same primers to Eurofins Genomics (Ebersberg, Germany).

The obtained 16S rRNA and *gyrB* sequences from bacterial isolates were deposited in the European Nucleotide Archive (ENA), with the following accession codes: LT891931 (16S isolate 8.1), LT891932 (16S isolate 8.2), LT891933 (16S isolate 9.1), LT891934 (16S isolate 9.2), LT891935 (16S isolate 19), LT891936 (16S isolate 47.1), LT891937 (16S isolate 47.2), LT891938 (16S isolate 49), LT891939 (16S isolate 3B), LT891940 (*gyrB* isolate 8.1), LT891941 (*gyrB* isolate 9.1) and LT891942 (*gyrB* isolate 9.2). Retrieved sequences were submitted to the taxonomic identification servers BLAST (16S ribosomal RNA database for 16S rRNA or Nucleotide collection nr/nt for *gyrB* sequences) and EzBioCloud (http://www.ezbiocloud.net/identify) (Yoon et al. 2017).

### Shake flask fermentations

Bacterial cultures from glycerol stocks were streaked on LB plates to obtain single colonies, and pre-cultures (25 mL) were started by inoculating a single colony of microorganism from the LB plate to M9 medium containing 10 g L^−1^ glucose. Cultivations were made in 250 mL shake flasks with 25 mL culture volume, placed in an orbital shaker operating at 180 rpm to uphold aerobic conditions at a temperature of 27 °C. After about 16 h of growth on glucose, cells were harvested and washed with sterile saline solution. Subsequently, the cells were transferred into other 250 mL flasks containing 50 mL M9 medium with 5 mM of either ferulate, *p*-coumarate, benzoate, syringate, vanillin, guaiacol, vanillate, 4-hydroxybenzoate, vanillyl alcohol or *cis,cis*-muconate. These flasks were also maintained at 27 °C and 180 rpm. The inoculation aimed to achieve an initial OD of around 0.1. In case the organism did not start growing until around 200 h, the experiments were repeated with an initial OD of 0.5. All shake flask experiments were conducted in duplicates.

Samples were withdrawn at regular intervals to measure the optical density (OD) spectrophotometrically at 620 nm. The samples were diluted to stay in the linear range of absorbance (0.03–0.3). After every sampling, the cells were removed by centrifugation at 12,300*g* for 3 min and the supernatants were filtered (0.2 µm pore size) and then stored at − 20 °C for UHPLC (Ultra High-Performance Liquid Chromatography) analysis.

### Determination of rates and yields

The measured optical density was recalculated to biomass concentration using a response factor of 0.62, determined from measured final biomass dry weight. Biomass yield [g(g)^−1^ or g(mmol)^−1^)] was calculated by subtracting the initial biomass supplied from the final biomass formed, divided by the total mass or amount of carbon substrate consumed. Specific growth rates were calculated by plotting the natural logarithm of biomass over time in its exponential phase. The specific substrate uptake rates were calculated for different time intervals from measured substrate consumption divided by the average biomass in that time interval. The overall specific substrate uptake rate was calculated as the average of determined specific substrate uptake rates during exponential growth.

### UHPLC analysis

A Waters Acquity UPLC system (Milford, USA) coupled with a photodiode array detector was used to perform the analysis of aromatic compounds. The type of column used for separation was a Waters Acquity UPLC BEH (Ethylene Bridged Hybrid) C18 column with an internal diameter of 2.1 mm, 100 mm length and 1.7 µm particle size. The temperature of the column was maintained at 47 °C. The mobile phases used were the binary solvent system consisting of fraction A (3% acetonitrile, 2% acetic acid, 95% MilliQ-water) and fraction B (85% acetonitrile, 2% acetic acid, 13% MilliQ-water). The sample injection volume was 2.5 µL.

The UPLC analysis method was adapted from Schwarz et al. (2009) with some modifications. To avoid high backpressure the flow rate was set to 0.6 mL/min. To compensate for the lower flow rate, the gradient time was extended to 11.5 min with: 0 min, 100% A; 5 min, 90% A; 7 min, 90% A; 11.5 min, 25% A. Finally, the column was washed with 100% B and 100% A for 5 min each. Analysis data was reviewed with Empower 3 Chromatography Data Software. Peaks were identified and quantified according to the area under the curve against their respective calibration standards.

### Orbitrap mass spectrometry

Orbitrap mass spectrometer was used to identify and confirm the excreted metabolic intermediates. Multiple stage tandem mass spectrometry (MS^n^) experiments were performed on LTQ Velos Pro system equipped with a heated electrospray ionization source (HESI) (Thermo Fisher Scientific, Bremen, Germany). Standards and samples were infused with a syringe at a flow rate of 20 μL min^−1^ using methanol as solvent. The HESI source was operated in negative mode using an ion source temperature of 50 °C, a capillary voltage of 2.5 kV and a sheath gas flow of rate of 10 arbitrary units. The capillary temperature was set to 275 °C. The MS^n^ experiments were performed using the Orbitrap mass analyzer at a resolution of 100,000 in a mass range of *m/z* 50–500. Collision induced dissociation was performed with a normalized collision energy of 35. The instrument was controlled by Xcalibur 2.2 software.

## Results

### Isolation and identification of bacterial strains from Baltic Sea sediments

A sample of sediments from a shore next to an old pulp and paper mill was analyzed in order to detect and isolate bacterial strains able to thrive in this type of heavily polluted environment. To this end, an enrichment culture was carried out in a minimal medium with different lignin-related aromatic substrates (ferulate, guaiacol and lignin-enriched pulping stream form softwood) as the sole source of carbon and energy. The bacterial colonies grown in these experiments were subsequently isolated by restreaking until obtaining pure cultures. The isolates found on guaiacol and softwood stream plates were tested for growth on ferulate, *p*-coumarate, benzoate, syringate and guaiacol in liquid shake flask cultures, however no growth was found (Additional file 1).

The isolates were identified by 16S rRNA amplification and sequencing. Sequences were submitted to two different identification servers, obtaining the candidates shown in Table 1. Among the nine strains isolated in this work, three belong to the genus *Pseudomonas*, and the most likely identity of the rest of isolates is *Lelliottia amnigena*, the actinobacterium *Rhodococcus erythropolis*, *Acinetobacter lwoffii*, and three isolates from the *Bacillaceae* family, namely *Lysinibacillus macroides*, *Bacillus licheniformis* and *Bacillus safensis*. Since three out of nine identified isolates were found to belong to the genus *Pseudomonas*, and considering the difficulty of classifying *Pseudomonas* species, a more accurate taxonomic classification was attempted by partial amplification and sequencing of the *gyrB* gene, which encodes a subunit of the DNA gyrase. The resulting sequences were aligned to the BLAST database, and the suggested candidates for each isolate are shown in Table 2.

**Table 2:** Identification of isolates from the genus *Pseudomonas* by partial *gyrB* sequencing

	Species	Query cover (%)	Identity (%)
Isolate 8.1	*P. syringae* pv. *delphinii*	99	88
	*P. syringae* RM12EL_22A	99	88
	*P. viridiflava* CFBP 1590	99	88
	*P. avellanae* CIP 105176 (Type strain)	99	88
Isolate 9.1	*P. fluorescens* PF02	99	99
	*P. fragi* ATCC 27362	99	99
	*P. deceptionensis* CECT 7677 (Type strain)	99	94
	*P. psychrophila* BS3667	99	94
Isolate 9.2	*P. mohnii* IPA-2 (Type strain)	98	97
	*P. moorei* RW10 (Type strain)	98	97
	*P. putida* P-2	98	97
	*P. vancouverensis* strain BS3656	98	95

As detailed in the following section, the isolate *Pseudomonas* sp. 9.1 exhibited the highest versatility in terms of utilization of lignin model compounds; therefore a more reliable assignment of this isolate to a particular species was performed. In order to achieve this, the partial 16S and *gyrB* sequences were manually aligned exclusively to those of the type strains from the closest candidates. As shown in Table 3, the highest scored type strains were *P. weihenstephanensis* according to 16S and *P. deceptionensis* according to *gyrB*. Taking into account the much higher discrimination power of the *gyrB* analysis (Yamamoto and Harayama 1995), isolate 9.1 was tentatively identified as a *Pseudomonas deceptionensis* strain, a species belonging to the *P. fluorescens* group and *P. fragi* subgroup according to Mulet et al. (2010).

**Table 3:** Assignment of *Pseudomonas* sp. isolate 9.1 to a species by alignment of 16S and *gyrB* sequences with those from the closest type strains

Type strain	16S rRNA		*gyrB*	
	Identity	Score	Identity	Score
*P. fluorescens*	98	2453	86	261
*P. fragi*	99	2536	92	974
*P. deceptionensis*	99	2560	*94*	*1050*
*P. psychrophila*	99	2536	94	1044
*P. weihenstephanensis*	*99*	*2575*	91	939

The best hits in the database are highlighted in italics

### Growth characterization of *Pseudomonas* sp. isolate 9.1 on lignin model compounds

Due to its fast growth on model compounds (ferulate, *p*-coumarate and benzoate) in M9 media during small scale cultivations (not shown), isolate 9.1 was selected for further characterization in the present study, and its growth on six lignin model compounds (ferulate, *p*-coumarate, benzoate, syringate, vanillin and guaiacol) was assessed.

**Fig. 2:**
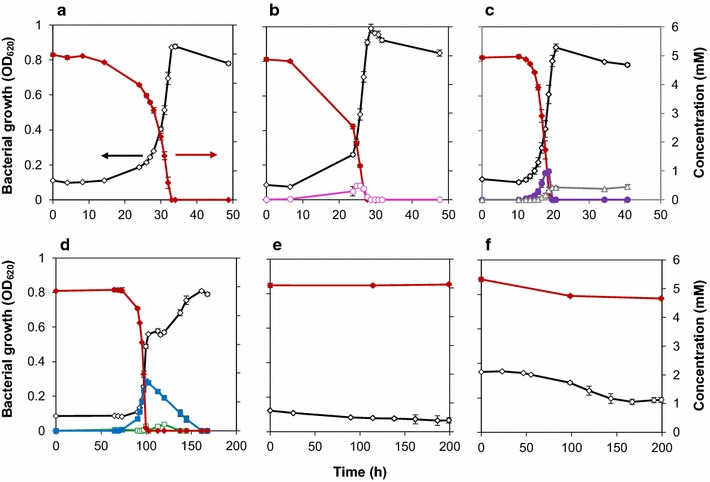
Growth of *Pseudomonas* sp. isolate 9.1 on **a** ferulate, **b** *p*-coumarate, **c** benzoate, **d** vanillin, **e** syringate and **f** guaiacol as the only source of carbon. Experiments were performed in duplicates and the standard deviations are displayed with an error bar. The OD and model compounds concentration (mM) are shown in black open diamond and red closed diamond respectively. The excreted intermediate compounds, if any, are also shown (Read from right axis). Pink open circle, 4-hydroxybenzoate; Violet closed circle, catechol; Grey open triangle, *cis*,*cis*-muconate; Blue closed square, vanillyl alcohol; green open square, vanillate

*Pseudomonas* sp. isolate 9.1 was able to grow on four out of the six model compounds and use them as a sole source of carbon and energy (Fig. 2). No growth was observed on syringate or guaiacol. There was a lag phase of about 10 h for growth on ferulate, *p*-coumarate, benzoate but a substantially longer lag phase of almost 100 h for vanillin. Several compounds were found to be excreted during growth on *p*-coumarate, benzoate and vanillin. The excreted compounds were detected by UHPLC and were identified by orbitrap MS–MS, corresponded to intermediate compounds of the respective funneling pathway. 4-hydroxybenzoate from *p*-coumarate, catechol from benzoate, vanillyl alcohol and vanillate from vanillin were subsequently consumed, except for *cis,cis*-muconate (from benzoate), which remained unconsumed even after 20 h (Fig. 2). A diauxic growth pattern was observed when vanillin was provided as a carbon source (Fig. 2d). During the first phase, there was growth on vanillin and (partial) conversion of vanillin to vanillyl alcohol. In the second phase, the produced vanillyl alcohol was consumed. A small amount of vanillate production and subsequent consumption was also observed (Fig. 2d).

### Growth of *Pseudomonas* sp. isolate 9.1 on excreted intermediate compounds

Further growth experiments were carried out on all the intermediate compounds found to be excreted during growth on the model compounds (4-hydroxybenzoate, vanillyl alcohol, vanillate and *cis,cis*-muconate) with the exception of catechol for which it was not possible to characterize growth since this compound was unstable in M9 medium.

**Fig. 3:**
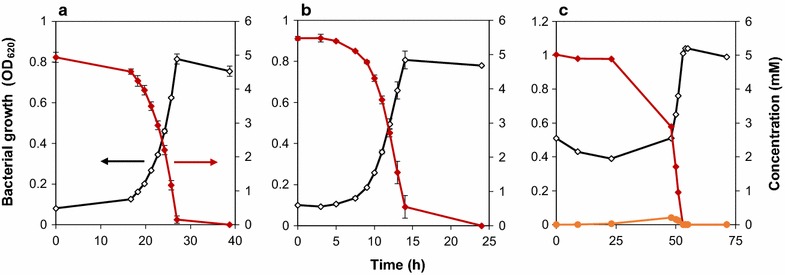
Growth of *Pseudomonas* sp. isolate 9.1 on **a** vanillate, **b** 4-HBA and **c** vanillyl alcohol as the only source of carbon. Experiments were performed in duplicates and the standard deviations are displayed with an error bar. One representative curve is shown for the growth and consumption on vanillyl alcohol. The OD and model compounds concentration (mM) are shown in black open diamond and red closed diamond respectively. The excreted intermediate vanillin is shown in orange closed circle (Read from right axis)

*Pseudomonas* sp. isolate 9.1 grew directly on 4-HBA without any lag phase (Fig. 3). The growth on vanillate had a substantial lag phase-around 15 h and the growth on vanillyl alcohol did not start until more than 50 h had elapsed (Fig. 3), and in one case (duplicate) not before 75 h. A small amount of excreted vanillin was detected during growth on vanillyl alcohol (Fig. 3c). Isolate 9.1 did not show any growth when *cis,cis*-muconate was given as a carbon source (data not shown). The compound was neither consumed nor converted into any other metabolite detected by UHPLC.

### Specific growth and uptake rates of *Pseudomonas* sp. isolate 9.1 on model compounds

The specific growth rates, uptake rates and yields were calculated for *Pseudomonas* sp. isolate 9.1 on all the lignin model compounds used for growth (Table 4). The biomass yields were in the range of 0.43–0.76 g_CDW_ g^−1^. The highest specific growth and uptake rates were observed for benzoate (0.3 h^−1^ and 4.2 mmol g_CDW_^−1^ h^−1^) whereas the lowest were for vanillyl alcohol (0.12 h^−1^ and 1.67 mmol g_CDW_^−1^ h^−1^) respectively. The specific conversion rate of *p*-coumarate (2.90 mmol g_CDW_^−1^ h^−1^) was slightly higher than for the 4-HBA intermediate (2.87 mmol g_CDW_^−1^ h^−1^). This may explain the excretion of 4-HBA during growth on *p*-coumarate. The uptake rate of vanillate and vanillyl alcohol added up roughly to the uptake rate of vanillin (Table 4). This is in line with a simultaneous uptake of vanillin via vanillate and a conversion of excess vanillin to vanillyl alcohol during growth on vanillin. As the uptake rate of vanillyl alcohol is lower than that of vanillin, the excretion of small amount of vanillin during growth on vanillyl alcohol was unexpected (Fig. 3). The conversion rate of ferulate and vanillate were almost equal (Table 4) and that could be the reason for intermittent trace excretion of vanillate during growth on ferulate (data not shown).

**Table 4:** Specific growth rates, yields and uptake rates of *Pseudomonas* sp. isolate 9.1 on lignin model compounds

Compounds	Specific growth rate (h^−1^)	Yield [g_CDW_ (mmol^−1^)] [g_CDW_ (g^−1^)]		Specific uptake rates [mmol (g_CDW_ h)^−1^]
Vanillin	0.15 ± 0.001	0.09 ± 0.001	0.62 ± 0.006	3.19 ± 0.065
Benzoate	0.30 ± 0.019	0.09 ± 0.001	0.76 ± 0.007	4.20 ± 0.155
*p*-Coumarate	0.29 ± 0.006	0.11 ± 0.005	0.68 ± 0.030	2.90 ± 0.197
Ferulate	0.18 ± 0.001	0.09 ± 0.001	0.48 ± 0.005	1.98 ± 0.025
Vanillate	0.18 ± 0.002	0.09 ± 0.004	0.55 ± 0.024	1.95 ± 0.032
4-HBA	0.29 ± 0.021	0.09 ± 0.001	0.65 ± 0.006	2.87 ± 0.360
Vanillyl alcohol	0.12 ± 0.004	0.06 ± 0.001	0.43 ± 0.006	1.67 ± 0.021

Experiments were performed in duplicates and the standard deviations are displayed

## Discussion

In the present study, a bacterial strain with extensive capacity for catabolism of aromatic compounds was isolated and physiologically characterized. The method of isolation, i.e. culture-dependent screening on either lignin or lignin-derived molecules from an interesting environment, is a widely used method to find lignin-utilizing prokaryotes (Tian et al. 2014) and proved successful also here.

Most of the bacterial isolates (five out of nine) from the enrichment cultures carried out in this study, belong to the *γ*-*proteobacteria* class, particularly to the genus *Pseudomonas*, the versatility of which to degrade and grow on lignin-related aromatic compounds and even technical lignins is widely acknowledged (Bandounas et al. 2011; Bugg and Rahmanpour 2015; Narbad and Gasson 1998). Three Gram-positive isolates from the *Bacillaceae* family were also detected by this growth-dependent method, namely *Bacillus licheniformis*, *B. safensis* and *Lysinibacillus macroides*. Many species of the genus *Bacillus* were previously found in deep-sea sediments in a similar screening carried out by Ohta et al. (2012), and most of them were also metabolically active toward lignin-related aromatic compounds. Finally, one *Rhodococcus erythropolis* strain, an actinomycete belonging to the high G+C Gram positive bacteria, was found as well. This is not surprising, since a number of rhodococci including *R. erythropolis* have been described among the most efficient bacterial lignin degraders (Ahmad et al. 2010) and are also able to metabolize the low-molecular weight compounds coming from lignin degradation (Taylor et al. 2012). Some of the bacterial isolates found in this work did not show ability to grow in liquid cultures with the model compounds utilized for their isolation (Additional file 1: Figs. S1 and S2). The initial growth on the screening plates might be due to the presence of intracellular storage compounds accumulated during the revitalization stage in rich medium.

Characterizing the aromatic metabolism, both in terms of catabolic pathways and the capacities of these pathways, is important to enable the design of efficient lignin conversion processes. The growth on low-molecular-weight lignin model compounds is relevant since the catabolism of such compounds is essential after, or along with, lignin mineralization (Bandounas et al. 2011). In the present study, six aromatic model compounds from the main different upper funneling pathways of lignin conversion were selected as carbon sources: vanillin, guaiacol and ferulate from the coniferyl branch; *p*-coumarate from the coumaryl branch; benzoate from the benzoyl branch and syringate from the sinapyl branch (Fig. 1). The coniferyl and coumaryl branches converge at protocatechuate (except for guaiacol which is converted to catechol), the benzoyl branch continues through catechol, whereas the sinapyl branch proceeds down to 3-*O*-methylgallic acid or even further to gallic acid. Protocatechuate and catechol enter the β-ketoadipate pathway ending with acetyl-CoA and succinyl-CoA, while 3-*O*-methylgallic acid/gallic acid undergoes ring cleavage and degrades to oxaloacetic acid and pyruvic acid. All these products either are the TCA (tricarboxylic acid) cycle intermediates or eventually enter the TCA cycle to promote cell growth. The isolate 9.1, tentatively identified as *P. deceptionensis* was able to take up four out of the six tested lignin model compounds as sole sources of carbon. The highest specific growth and uptake rates were measured for benzoate (Table 4), indicating that the benzoyl branch is the fastest of the upper funneling pathways in this isolate. The second fastest uptake occurred in the *p*-coumaryl branch with uptake rates of *p*-coumarate and 4-HBA around 2.9 mmol g_CDW_^−1^ h^−1^. The coniferyl branch operated at a somewhat lower flux rate, with uptake rates of ferulate, vanillate and vanillyl alcohol lower than 2 mmol g_CDW_^−1^ h^−1^. This might be due to the presence of phenylmethyl ether linkage in the coniferyl branch, which is comparatively difficult to breakdown (Okamura-Abe et al. 2016). No growth was obtained for syringate and guaiacol. The difficulty in metabolizing highly methoxylated substrates such as those present in the syringyl branch is most likely due to the complex enzymatic systems required for its demethylation. The *O*-demethylase systems for dimethoxylated compounds are well described in the case of *Sphingobium* sp. SYK-6, *Acetobacterium woodii*, *Acetobacterium dehalogenans* and *Moorella thermoacetica* (Berman and Frazer 1992; Kaufmann et al. 1998; Naidu and Ragsdale 2001). They consist of two methyl transferases which convey the methyl groups from the aromatic substrate (e.g. veratrol, syringate) to an accepting cofactor like tetrahydrofolate (H_4_F), often with the involvement of a corrinoid protein. These systems usually show relatively high substrate specificity, and also require continuous supply and enzymatic recycling of the cofactor; moreover, when a corrinoid protein is involved, spontaneous oxidation of its cobalt group can take place, and ATP-consuming enzymatic regeneration of this oxidized catalyst will be necessary (Siebert et al. 2005). Additionally, the demethylation reactions can result in the formation of formaldehyde or formic acid (Masai et al. 2007; Overhage et al. 1999), toxic intermediates that require further processing (Hibi et al. 2005), which can contribute to the slower metabolism of these compounds (Additional file 1: Figs. S3 and S4).

There was a short lag phase of approximately 15 h for ferulate, *p*-coumarate and benzoate (Fig. 2), necessary for adaption to the new carbon source and expression of required enzymes after prior growth on glucose (Kurosawa et al. 2015). A much longer lag phase, around 100 h, for growth on vanillin (Fig. 2) and no growth on syringate for 200 h, was observed. The antimicrobial effect of both compounds is well documented for many organisms. The concentration of vanillin and syringate required to inhibit the growth the *Escherichia coli* LY01 by 50% is 0.9 and 1.6 g/L respectively (Zaldivar et al. 1999). The mechanism of inhibition can be either bacteriostatic or bactericidal. The increased uptake of nucleic acid stain upon exposure of *E. coli* to vanillin suggests that the integrity and stability of the cell membrane was affected, leading to cell lysis (Fitzgerald et al. 2004). In another study, the addition of syringic acid at the concentration of IC_80_ (80% inhibitory concentration), proved to destroy the membrane integrity by increasing membrane leakage by three fold (Zaldivar and Ingram 1999). However, in our case, no major cell lysis (i.e. no decrease in OD) was observed when exposed to vanillin or syringate, which suggests that the inhibition was bacteriostatic. A quite long lag phase before growth on vanillin, might explain the time required by the organism to repair the cell damage and overcome the inhibition (Rolfe et al. 2012).

Interestingly, *Pseudomonas* sp. isolate 9.1 while growing on some model compounds excreted certain pathway intermediates. This contrasts to previous observations with *P. putida* strains (Ravi et al. 2017). It appears that isolate 9.1 is not regulated to maintain the same flux throughout the pathway to the same extent as *P. putida*. With the exception of *cis,cis*-muconate, all the excreted intermediates were later consumed, thereby enabling the complete utilization of the carbon source, although in a di- or tri-auxic growth pattern. *Cis,cis*-muconate and catechol were excreted during growth on benzoate, but while catechol was subsequently taken up, *cis,cis*-muconate remained unconsumed (Fig. 2c). Possibly *Pseudomonas* sp. isolate 9.1 lacks or has a defective version of the gene encoding the transporter involved in the uptake of *cis,cis*-muconate, which in *Acinetobacter* and other *Pseudomonas* strains has been identified as *mucK* (Williams and Shaw 1997), a member of the family 15 of MFS transporters (Pao et al. 1998). It is not clear how the accumulated *cis*,*cis*-muconate is excreted outside of the cell without a functional transporter, but there may be other transporters able to move this compound across the cell membrane with lower specificity. *Cis,cis*-muconate can be used as a precursor to adipic acid, one of the monomers in nylon-6,6 (Vardon et al. 2015) and has therefore attracted quite some interest. Bioconversion of benzoate to *cis,cis*-muconate was explored in *P. putida* KT2440-JD1 (van Duuren et al. 2012). Also in this *P. putida* strain, the maximum uptake rate of benzoate was higher than the production rate of *cis,cis*-muconate, which is the reason for the excretion of benzoate derivatives in the cells. Excessive uptake of benzoate is also a reason for the excretion of catechol, which reduces the expression of the *ben* operon leading to the decreased conversion of benzoate to *cis,cis*-muconate (van Duuren et al. 2012).

Accumulation of catechol in the culture media might be toxic to the organism since catechol is unstable at pH 7 and produces reactive oxygen species. The oxidized species spontaneously form colored polymers (Subramanian and Worden 1993; Sudarsan et al. 2016; Yang et al. 2014), which give experimental problems to study catechol as a sole source of carbon in growth experiments, also in the present work.

Somewhat unexpectedly, vanillyl alcohol was excreted during growth of *Pseudomonas* sp. isolate 9.1 on vanillin (Fig. 2 vanillin). As vanillin is a potential inhibitor to many microorganisms, the conversion of vanillin to less toxic intermediates is quite common (Gallage and Møller 2015). In bacteria, especially pseudomonads, vanillin is normally converted to vanillate (Graf and Altenbuchner 2014; Ravi et al. 2017). However, under growing conditions, *P. fluorescens* B56 was able to convert vanillin to vanillate with a small amount of vanillyl alcohol being excreted (Asm et al. 1988). In addition, a genetically modified *Pseudomonas* strain (vanillin dehydrogenase deletion mutant) was able to produce vanillyl alcohol via ferulic acid and vanillin, when eugenol was given as a sole carbon source (Takasago Perfumery Co Ltd 1993). Although the intention of genetic modification was to produce vanillin from eugenol, the strain further converted some amount of vanillin to vanillyl alcohol, which proves the existence of such activity under certain conditions.

The conversion of vanillin to vanillyl alcohol is more common as a part of the detoxification process in eukaryotes such as *Phanerochaete chrysosporium* or *Saccharomyces cerevisiae*, (Nguyen et al. 2015; Stentelaire et al. 1997). The uptake rate of vanillin by isolate 9.1 was 3.19 mmol g_CDW_^−1^ h^−1^, whereas the uptake of vanillate was only 1.95 mmol g_CDW_^−1^ h^−1^. The excess uptake of vanillin was apparently channeled to vanillyl alcohol, which was subsequently giving rise to a diauxic growth pattern.

Another observation was that *Pseudomonas sp.* isolate 9.1 had a relatively stable stationary phase after the carbon source was depleted (Figs. 2, 3). This is in contrast to our previous work with *P. putida* KT2440, in which case there was a rapid decrease in the biomass concentration directly after the depletion of the carbon source without a stable stationary phase (Ravi et al. 2017).

In conclusion, the isolate 9.1, tentatively identified as *Pseudomonas deceptionensis* based on *gyrB* alignment, appears to possess most of the major upper funneling pathways necessary for the conversion of lignin-related aromatic compounds. Growth on model compounds does not mean that the organism will necessarily convert lignin or depolymerized lignin. However, it is a prerequisite for such conversion to occur.

Further work will show if this isolate or other bacterial strains are able to metabolize or partially convert different types of depolymerized lignin, which is necessary for the establishment of efficient processes in the future biorefineries, paving the way for a complete exploitation of the forestry and agricultural resources.

## Additional file

**Additional file 1.**  Characterization of bacterial isolates on lignin model compounds.

## Abbreviations

rRNA: ribosomal ribonucleic acid
CDW: cell dry weight
NaCl: sodium chloride
PCR: polymerase chain reaction
bp: base pairs
LB: Lysogeny Broth
rpm: revolutions per minute
OD: optical density
UHPLC: ultra high performance liquid chromatography
BEH: ethylene bridged hybrid
MS^n^: multiple stage tandem mass spectrometry
HESI: heated electrospray ionization
m/z: mass-to-charge ratio
4-HBA: 4-hydroxybenzoic acid
G+C: guanine and cytosine content
TCA cycle: tricarboxylic acids cycle
H^4^F: tetrahydrofolate
MFS transporters: major facilitator superfamily transporters
