# Supplementary material for: Biological conversion of aromatic monolignol compounds by a Pseudomonas isolate from sediments of the Baltic Sea
Source: AMB Express. 2018 Mar 2;8:32. doi: 10.1186/s13568-018-0563-x (PMC5834416; doi:10.1186/s13568-018-0563-x)
Supplement: Supplementary file 1 — Additional file 1. Characterization of bacterial isolates on lignin model compounds. [file 13568_2018_563_MOESM1_ESM.pdf]

AMB Express

Title

Biological conversion of aromatic monolignol compounds by a *Pseudomonas* isolate from sediments of the Baltic Sea

Authors

Krithika Ravi[1], Javier García-Hidalgo[2*], Matthias Nöbel[1], Marie F Gorwa-Grauslund[2], and Gunnar Lidén[1]

[1]Department of Chemical Engineering, Lund University, P.O. Box 124, SE-221 00 Lund, Sweden

[2]Department of Chemistry, Applied Microbiology, Lund University, P.O. Box 124, SE-221 00 Lund, Sweden

[*]Corresponding author: javier.garcia_hidalgo@tmb.lth.se

Additional file 1

Characterization of bacterial isolates on lignin model compounds

The bacterial species isolated from guaiacol (isolate 47.1 and 47.2, tentatively identified as *Acinetobacter lwoffii* and *Lysinibacillus macroides* respectively) and softwood lignin streams (isolate 49 and 3B, tentatively identified as *Bacillus licheniformis* and *B. safensis*) were tested for growth on 5 mM each of ferulate, *p*-coumarate, benzoate, syringate and guaiacol in liquid shake flask cultures. No growth was observed for any of the organisms on model compounds (Fig. S1, S2, S3 and S4), except for *A. lwoffii* on benzoate which was consumed within 20 h (Fig. S1). As previously reported (Koschorreck et al. 2008), the *Bacillus* strains due to its laccase activity, converted *p*-coumaric and ferulic acids (Fig. S3 and S4) most likely into dimers and trimers, which were found as new peaks in the UHPLC analysis. None of the strains isolated from guaiacol were able to grow with guaiacol as the only carbon source in liquid M9 medium. Possibly, the observed growth on agar plates was initially due to carbon sources in the form of intracellular storage compounds accumulated in previous stages.

Koschorreck K, Richter SM, Ene AB, Roduner E, Schmid RD, Urlacher VB (2008) Cloning and characterization of a new laccase from Bacillus licheniformis catalyzing dimerization of phenolic acids. Applied Microbiology and Biotechnology 79(2):217-224 doi:10.1007/s00253-008-1417-2

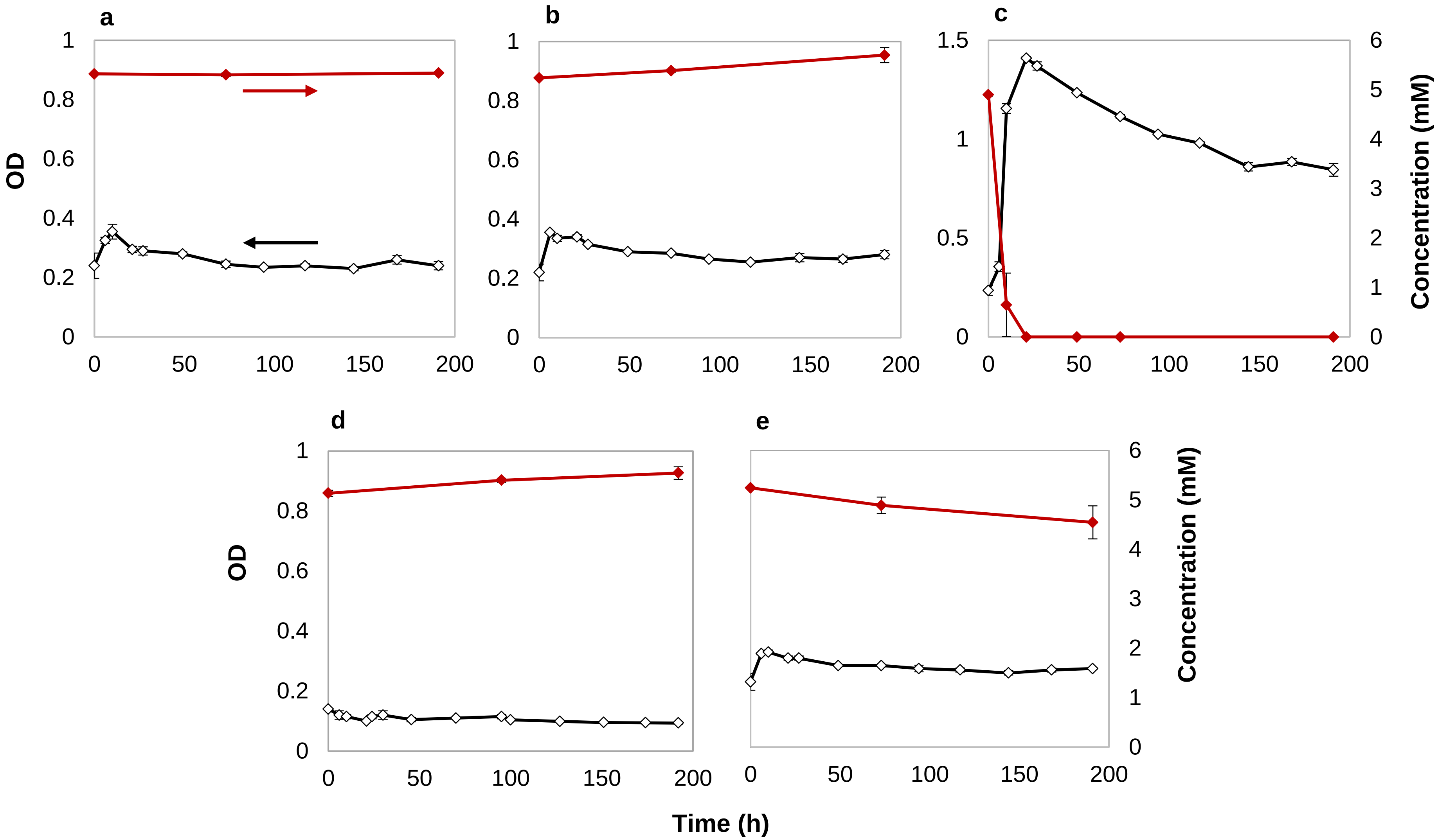

Fig. S1. Growth of *Acinetobacter lwoffii* (isolate 47.1) on (a) ferulate, (b) *p*-coumarate, (c) benzoate, (d) syringate and (e) guaiacol as the only source of carbon. The OD and model compounds concentration (mM) are shown in black open diamond and red closed diamond respectively.

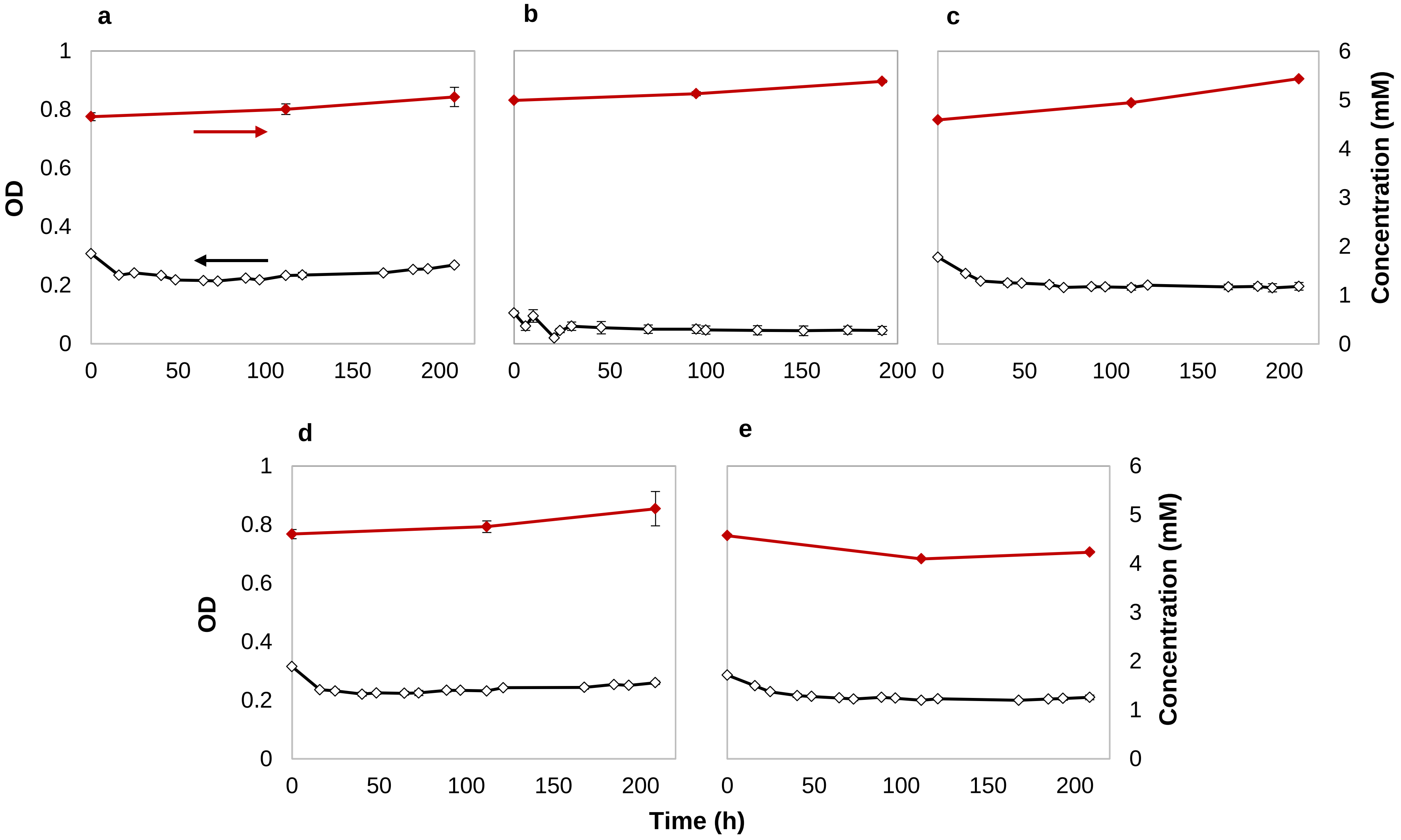

Fig. S2. Growth of *Lysinibacillus macroides* (isolate 47.2) on (a) ferulate, (b) *p*-coumarate, (c) benzoate, (d) syringate and (e) guaiacol as the only source of carbon. The OD and model compounds concentration (mM) are shown in black open diamond and red closed diamond respectively.

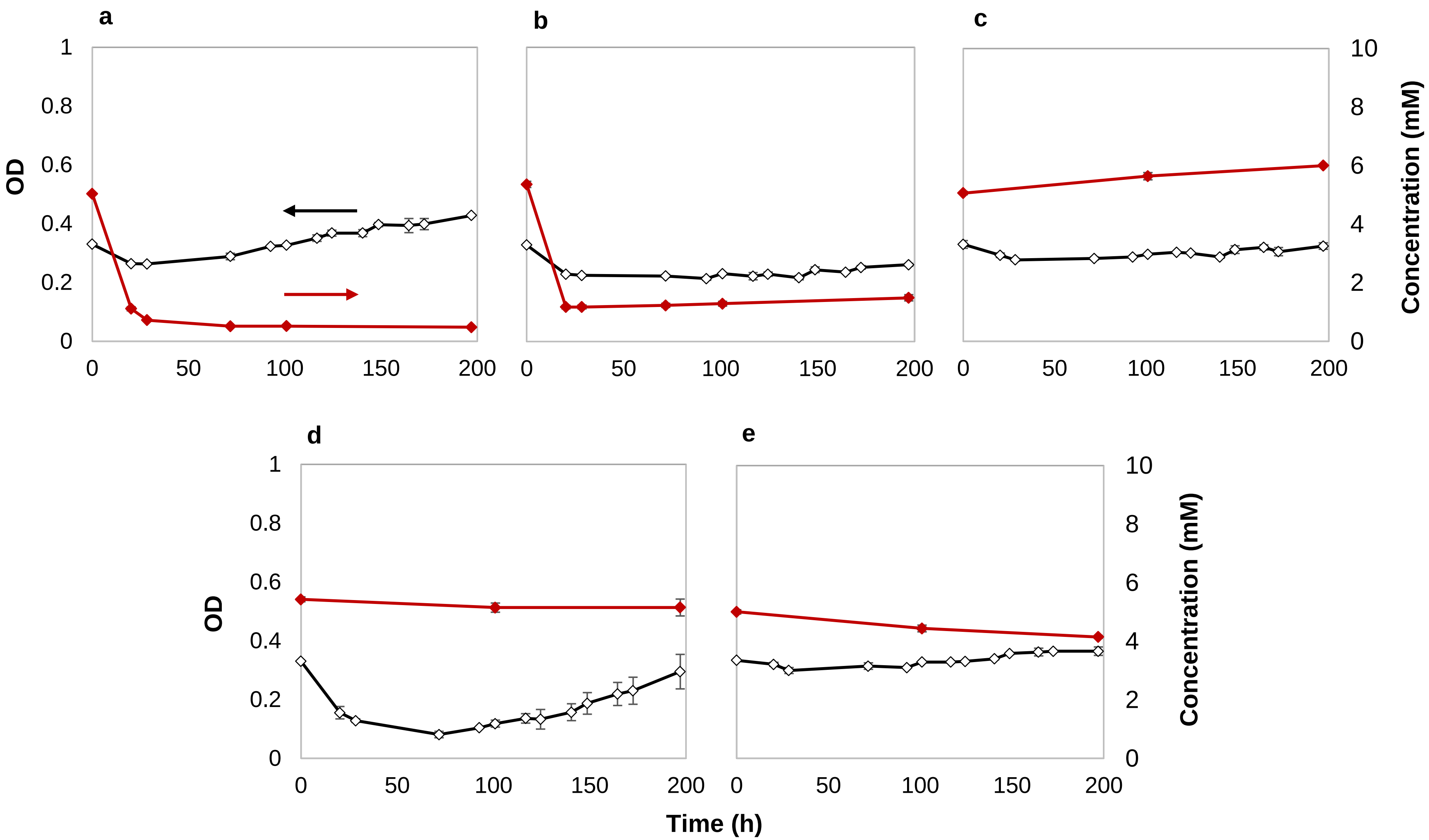

Fig. S3. Growth of *Bacillus licheniformis* (isolate 49) on (a) ferulate, (b) *p*-coumarate, (c) benzoate, (d) syringate and (e) guaiacol as the only source of carbon. The OD and model compounds concentration (mM) are shown in black open diamond and red closed diamond respectively.

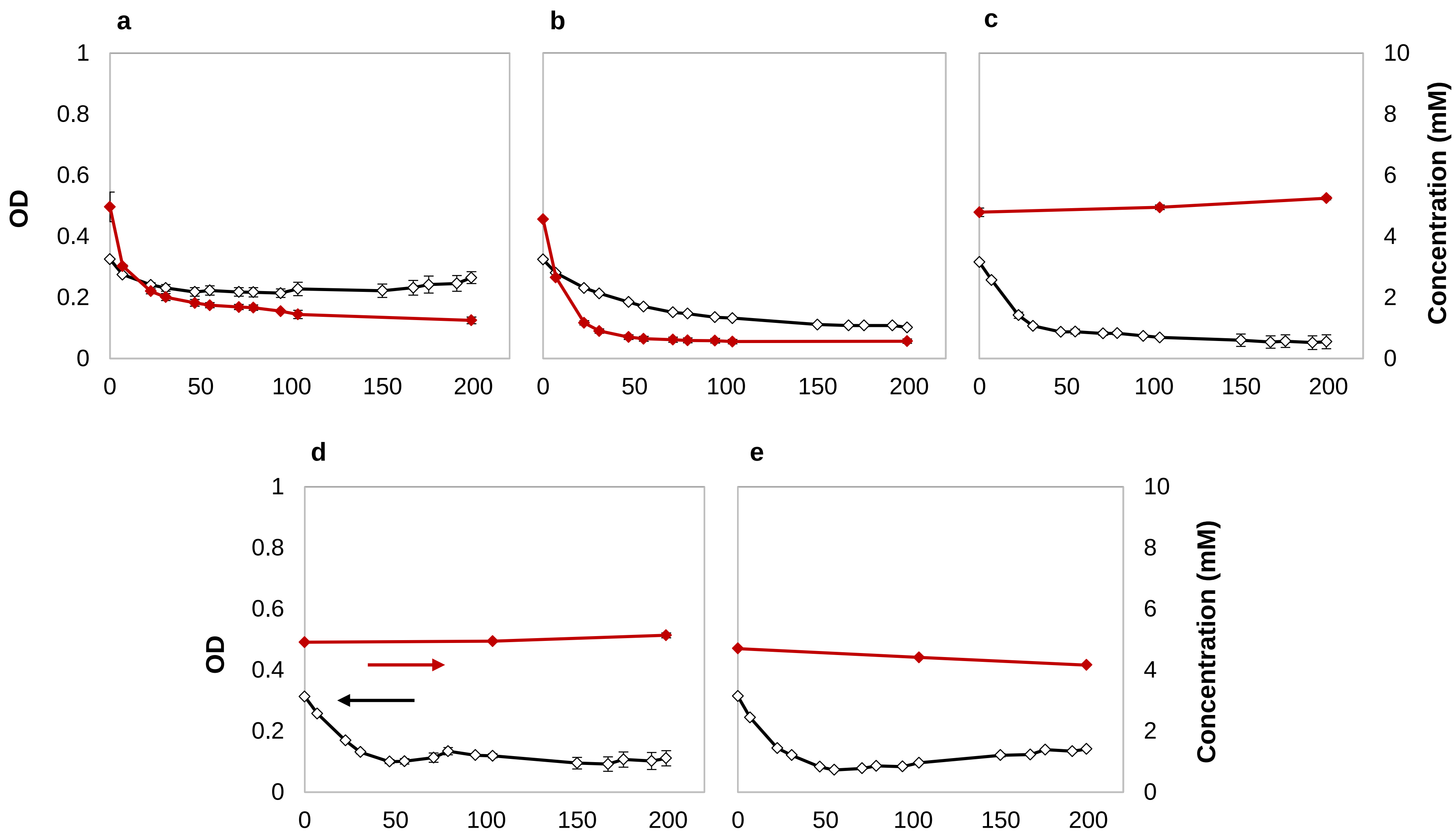

Fig. S4. Growth of *Bacillus safensis* (isolate 3B) on (a) ferulate, (b) *p*-coumarate, (c) benzoate, (d) syringate and (e) guaiacol as the only source of carbon. The OD and model compounds concentration (mM) are shown in black open diamond and red closed diamond respectively.
